# DNA Methylation Signatures of Bone Metabolism in Osteoporosis and Osteoarthritis Aging-Related Diseases: An Updated Review

**DOI:** 10.3390/ijms22084244

**Published:** 2021-04-19

**Authors:** Virginia Veronica Visconti, Ida Cariati, Simona Fittipaldi, Riccardo Iundusi, Elena Gasbarra, Umberto Tarantino, Annalisa Botta

**Affiliations:** 1 Department of Biomedicine and Prevention, University of Rome “Tor Vergata”, Via Montpellier 1, 00133 Rome, Italy; virginia.veronica.visconti@uniroma2.it (V.V.V.); ida.cariati@uniroma2.it (I.C.); simona.fittipaldi@uniroma2.it (S.F.); botta@med.uniroma2.it (A.B.); 2 Department of Orthopaedics and Traumatology, “Policlinico Tor Vergata” Foundation, Viale Oxford 81, 00133 Rome, Italy; riccardo.iundusi@uniroma2.it (R.I.); gasbarra@med.uniroma2.it (E.G.); 3 Department of Clinical Science and Translational Medicine, University of Rome “Tor Vergata”, Via Montpellier 1, 00133 Rome, Italy

**Keywords:** epigenetics, DNA methylation, bone metabolism, osteoporosis, osteoarthritis, aging-related diseases

## Abstract

DNA methylation is one of the most studied epigenetic mechanisms that play a pivotal role in regulating gene expression. The epigenetic component is strongly involved in aging-bone diseases, such as osteoporosis and osteoarthritis. Both are complex multi-factorial late-onset disorders that represent a globally widespread health problem, highlighting a crucial point of investigations in many scientific studies. In recent years, new findings on the role of DNA methylation in the pathogenesis of aging-bone diseases have emerged. The aim of this systematic review is to update knowledge in the field of DNA methylation associated with osteoporosis and osteoarthritis, focusing on the specific tissues involved in both pathological conditions.

## 1. Introduction

**Figure 1:**
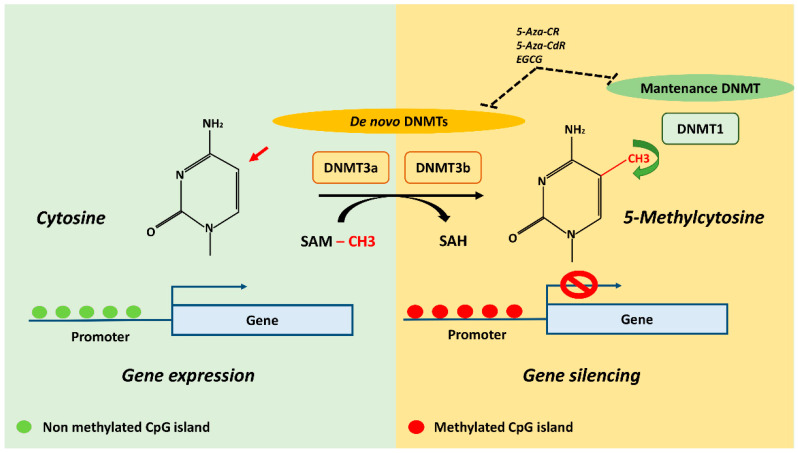
DNA methylation mechanism and potential DNMT inhibitors (DNMTi). The transfer of methyl (CH3) group to the C5 position of the cytosine to create 5-methylcytosine (5mC) is actuated by DNMTs which catalyze the addition by sequestering CH3 group from SAM methyl donor. DNMT3a and DNMT3b establish initial methylation patterns and DNMT1 maintain the already established methylation patterns. The CpG islands represents the richest regions of GC sites which are usually located in gene promoters and 5′ regulatory regions, and its methylation is generally associated with gene silencing. Demethylating agents 5-Aza-CR, 5-Aza-CdR and EGCG are known to inhibit DNMT3a, DNMT3b and DNMT1 activity. DNMTs, DNA methyltransferases; SAM, S-adenosylmethionine; SAH, S-adenosylhomocysteine; 5-Aza-CR, 5-Azacytidine; 5-Aza-CdR, 5-aza-2′-deoxycitidine; EGCG, epigallocatechin-3-gallate.

DNA methylation is an inheritable change in gene activity or function which does not change the DNA sequence and plays a key role in regulating gene expression by different mechanisms [1,2,3]. In mammals, this epigenetic mechanism is based on the transfer of a methyl (CH3) group to the C5 position of the cytosine to create 5-methylcytosine (5mC) [4]. The transfer is actuated by a class of enzymes called DNA Methyltransferases (DNMTs) which catalyze the addition of a CH3 group by sequestering it from S-adenyl Methionine (SAM) methyl donor [5] (Figure 1).

DNMTs are divided into two categories: de novo DNMTs, which establish initial methylation patterns (DNA Methyltransferase 3a, DNMT3a; DNA Methyltransferase 3b, DNMT3b) and maintenance DNMTs, which maintain the already established methylation signature (DNA (cytosine-5-)-Methyltransferase 1, DNMT1) [6]. DNA methylation is found in different sequence contexts such as intergenic regions, CpG islands and gene body. Intergenic regions are represented by transposable and viral elements, which are usually inactivated by DNA methylation to repress the expression of potentially harmful genetic elements. Indeed, the replication and insertion of these factors can lead to gene disruption and DNA mutation [4]. CpG islands represents the richest regions of GC sites, accounting for about 1–2% of the entire genome and are usually located in gene promoters and 5′ regulatory regions [7]. DNA methylation is a mechanism capable of regulating gene expression through the recruitment of proteins involved in gene repression or by physically impeding the binding of transcription factors to DNA [4]. Finally, the gene body also contains DNA methylation sites, but the mechanisms by which these contribute to the regulation of gene expression are still being explored. However, transcribed regions of genes could be strongly methylated, leading to results being positively correlated with gene expression levels. DNA methylation in these regions could silence alternative promoters, retrotransposon elements and other functional elements to maintain transcription efficiency [8].

**Figure 2:**
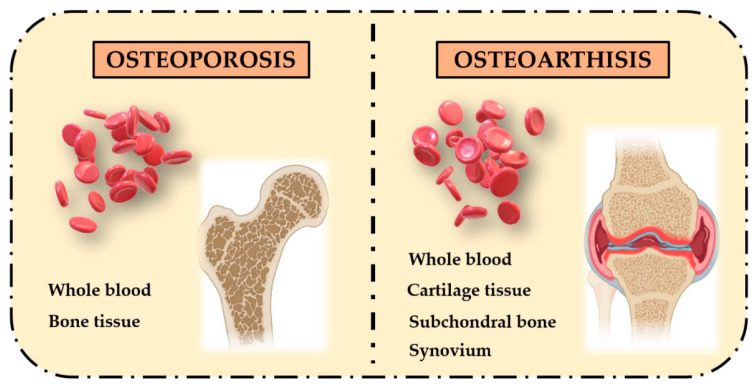
Different affected tissues in OP and OA bone-related phenotypes. OP pathogenesis mainly involves bone tissue, while OA pathogenesis involves multiple tissues, including cartilage, subchondral bone and synovium.

In the last years, dynamic DNA methylation signatures in specific CpG loci are strongly considered as biomarkers in aging and age-related diseases, representing an important clinical goal for diagnosis, prognosis and prediction of response to therapies [9]. Among age-related diseases, bone-metabolism diseases, such as osteoporosis (OP) and osteoarthritis (OA), are a widespread health problem worldwide and several studies investigated the association between their pathogenic mechanisms and DNA methylation patterns. These are complex late-onset multifactorial disorders with a strong epigenetic component which affect bones in different ways [10], leading to an imbalance between osteoblasts’ bone formation and osteoclasts’ bone resorption. Recent studies exploring global and gene-specific methylation profiles in whole blood identified epigenetic signatures to be significantly associated with OP and OA disease progression, which could represent non-invasive prognostic biomarkers. However, tissues involved in the pathogenesis of both diseases are different: OP leads mostly to bone tissue alterations whereas in OA, multiple tissues, including cartilage, subchondral bone and synovial tissue, are impaired (Figure 2). 

The main aim of this systematic review is to provide an overview of the role of DNA methylation in tissues specifically involved in both diseases by describing studies conducted in vitro and ex vivo in OP and OA cells and patients. The final goal is to elucidate the impact of this epigenetic regulatory mechanism in the pathogenesis of OP and OA aging-related bone diseases. 

## 2. Literature Search Strategy

An independent literature search was conducted across the international bibliographic web databases PubMed, Medline, and Web of Science. The investigation to identify papers relevant to our systematic review was carried out until February 2021. Papers concerning the association between DNA methylation signatures in age-related bone metabolism diseases, such as OP and OA, were selected. The search strategy was based on a combination of the following keywords: “methylation”, “epigenetics”, “aging”, “osteoporosis”, “osteoarthritis”, “osteoblasts”, “bone”, “bone fragility” and “fragility fractures”. Furthermore, a manual search was conducted for references of relevant systematic reviews and original articles. The search process was carried out on a worldwide basis, without excluding specific geographical areas or different ethnic groups. Language and species filters were then applied to the list of output results to eliminate non-English articles and animal studies. Case reports, abstracts, short communications, letters to the editor, dissertations and studies lacking case and control adequate numbers were also excluded. The overall data reported in this review were extrapolated by consulting a total of 96 publications.

## 3. DNA Methylation Pattern in Osteoporosis 

Osteoporosis, the most common bone disease, is characterized by systemic microarchitecture impairment and reduced bone mass, resulting in increased bone fragility and risk of fractures [11]. Since genetic factors as well as epigenetic modifications play a key role in bone homeostasis, many scientific papers explored both mechanisms in OP pathogenesis [12]. The majority of epigenetic studies in osteoporotic subjects are focused on noncoding RNAs (either miRNAs and lncRNAs) as post transcriptional regulators of target mRNAs [13,14,15]. On the other hand, only a few papers analyzed the role of DNA methylation in OP pathogenesis. In this section, both systemic (whole blood) and tissue-specific (bone tissue) DNA methylation studies are summarized (Table 1). Data collection includes single genes and epigenome-wide studies, with the aim of providing an updated overview on the DNA methylation mechanisms regulating the expression of key genes involved in OP pathogenesis.

### 3.1. Whole Blood 

One of the main challenges in the identification of epigenetic factors in OP is the difficulty in obtaining bone tissue biopsies from patients. Therefore, researchers have focused their attention on the analysis of DNA methylation signatures in blood, which can be considered as a valid non-invasive alternative. One of the first epigenetic studies in OP patients analyzed the methylation of Alu elements in blood [16], which are interspatial repetitive DNA elements that are gradually hypomethylated with aging [26]. Specifically, Jintaridth et al. used Combined Bisulfite Restriction Analysis (COBRA) on a study cohort of 323 postmenopausal women to investigate the possible association between age-related epigenetic variation in Alu elements and bone density [16]. Data obtained showed that Alu hypomethylation in blood cells is positively correlated with both older age and lower bone mass density, confirming its association with OP metabolism. This evidence suggests that Alu methylation levels may be an indicator of age-related diseases, paving the way for further studies to clarify the roles of epigenetic modifications in OP phenotypes [16]. One of the key factors involved in OP pathogenesis is represented by Bone Morphogenetic Proteins (BMPs), as multifunctional growth factors which promote bone and cartilage formation [27]. These molecules belong to the Transforming Growth Factor-β (TGF-β) family and are able to regulate several biological processes linked to cell proliferation, differentiation, homeostasis and regeneration [28]. In addition, BMPs provide morphogenetic signals for skeletal development during embryogenesis and are responsible for adult fracture healing, triggering a cascade of cellular events associated with embryonic bone formation [29]. A DNA methylation analysis of a CpG island located within *Bone Morphogenetic Protein 2 (BMP2)* promoter region comprising 14 CpG sites revealed interesting data on this key bone factor associated with OP pathogenesis. Whole blood analysis in individuals of Asian Indian Origin reported a frequency of methylated “C” at -267th bps from Transcriptional Start Sites (TSS) of 0.7 in 24 OP patients and 0.25 in 24 healthy subjects. The involvement of DNA methylation mechanism in regulating *BMP2* gene expression was confirmed by its lower expression levels and transcriptional activity in OP subjects. This epigenetic regulatory mechanism could interfere with the expression of downstream genes involved in the BMP2 signaling pathway, resulting in altered osteoblastogenesis and bone formation [17]. Another gene-specific DNA methylation study investigated the epigenetic role of the *cTBC1 Domain Family Member 8* gene *(TBC1D8)*, previously identified by a genome-wide association study as a novel susceptibility locus for OP-related traits [18]. The *TBC1D8* gene is one of the human protein molecular activity regulatory genes and represents an activator of the Guanosine Triphosphate (GTP) enzyme. Epigenetic analysis performed in a cohort of postmenopausal Chinese women revealed decreased methylation of the *TBCF1D8* gene promoter CpG island in 30 OP patients (n = 30) compared to healthy subjects (n = 30), suggesting new avenues of investigation into the functional role of this gene in OP pathogenesis [18]. A major contribution to this field is provided by Epigenome-Wide DNA Association Studies (EWAS), which examine a genome-wide set of quantifiable epigenetic marks, including DNA methylation. An Illumina Infinium human methylation 450 K analysis performed by Cheishvili et al. on whole blood DNA from 22 healthy and 22 postmenopausal OP women identified 13 most significant genes. Only five of these play a role in bone biology: *Zinc Finger Protein 267* (*ZNF267*), *Actin Binding LIM Protein Family Member 2* (*ABLIM2*), *Ras Homolog Family Member J* (*RHOJ*), *Cyclin-Dependent Kinase-Like 5* (*CDKL5*) and *Programmed Cell Death 1* (*PDCD1*). *ZNF267* displayed statistically significant hypomethylation, while *ABLIM2*, *RHOJ*, *CDKL5* and *PDCD1* showed hypermethylation in OP patients compared to healthy controls [19]. These results are discordant with another DNA methylation analysis performed by Infinium Human-Methylation450 BeadChips in peripheral blood from patients with primary OP, which did not reveal any alterations in the disease group compared to healthy subjects. The same research team of Fernandez–Rebollo et al. also investigated the existence of a potential increase in age-associated DNA methylation patterns in primary OP with no statistically significant associations [20]. These data suggest that the role of DNA methylation in OP pathogenesis is still full of gaps and further studies are needed to better understand the contribution of this epigenetic mechanism in modulating bone metabolism in OP patients.

### 3.2. Bone Tissue 

Bone is the main actor involved in OP pathogenesis and several recent studies show the involvement of epigenetic mechanisms, such as DNA methylation, in modulating factors associated with bone metabolism. A negative regulator in bone formation process is Sclerostin (SOST), a small protein secreted by mature osteocytes that plays the role of inhibiting the Wnt/β-catenin signaling pathway [30]. Reppe and colleagues suggested the existence of an epigenetic regulation mechanism for SOST expression in bone biopsies from postmenopausal OP women, revealing an increased CpG methylation in *SOST* gene promoter region in 27 patients compared to 36 healthy subjects. It was also observed that the increased methylation level of *SOST* in patients with low Bone Mineral Density (BMD) was functionally associated with the reduction of bone *SOST* mRNA and SOST serum levels [21]. The inhibitory role of SOST in bone formation was also confirmed by the Delgado–Calle team, who in a previous study showed hypomethylation in the *SOST* promoter region in human osteocytes [31]. These data suggest that the increased methylation of *SOST* in OP patients may be a compensatory counteracting mechanism leading to lower serum sclerostin levels, which reduces inhibition of Wnt signaling and promotes bone formation. Recently, another study performed on 12 postmenopausal OP women with bone fractures and 8 healthy subjects confirmed the increased methylation levels of two CpG islands located in the *SOST* promoter region. This result is reinforced by chromatin immunoprecipitation analysis that correlated the increased methylation with reduced binding of the activators factor Osterix (SP7), Runt-related transcription factor 2 (RUNX2) and Estrogen Receptor (ER) to the *SOST* promoter in OP patients [22]. Moreover, bisulfite sequencing of *SOST* promoter region revealed a pattern of hypermethylation in both the 16 osteoporotic fracture (OPF) bone tissue and 16 non-osteoporotic fracture (non-OPF) bone tissue groups [23]. Nevertheless, the methylation ratio was lower in the OPF compared to the non-OPF group. To confirm the pivotal role played by bone SOST in OP pathogenesis, Reppe et al. combined transcript profiling with DNA methylation analyses in bone biopsies. Four transcripts inhibiting bone formation were identified: *SOST*, *Dickkopf WNT Signaling Pathway Inhibitor 1* (*DKK1*) and *WNT Inhibitory Factor 1* (*WIF1*), encoding for proteins targeting the Wnt pathway and signaling. Their results showed an organized regulation of these transcripts, which also correlated with the highest number of methylated CpG sites [13]. Another key factor in the regulation of bone resorption is the RANKL/OPG ratio. The binding of Receptor Activator of Nuclear Factor-κB (RANK) with its receptor RANK ligand (RANKL), stimulates pre-osteoclast fusion, promoting osteoclast adhesion to bone and preventing apoptosis. In contrast, Osteoprotegerin (OPG) inhibits RANKL binding and promotes the apoptotic process, leading to a reduction in the osteoclasts number. The methylation analysis of *RANKL* and *OPG* promoter regions in OP bone tissue showed an inverse correlation between the expression level and the methylation status of these genes. In fact, RANKL expression in the OP group was significantly higher than in healthy controls, in accordance with a lower CpG methylation status of the promoter. Conversely, reduced *OPG* gene expression and high methylation level were found in the OP group [24]. Finally, given the important role of cancellous bone in bone development [24], the research also focused on EWAS in this tissue derived from patients with postmenopausal osteoporosis. This microarray-based analysis identified 13 genes differently methylated in OP patients, reported in Table 1 to be involved in five important bone-related signaling pathways (calcium; Cyclic Guanosine Phospho-Protein Kinase G, cGMP-PKG; endocytosis; Ras-Associated Protein-1, Rap1; Adenosine Activated Protein Kinase, AMPK) [25]. Undoubtedly, genome-wide studies represent an invaluable tool in understanding epigenetic mechanisms, although functional validation are needed to confirm novel identified DNA methylation patterns that may contribute to OP development.

## 4. DNA Methylation Pattern in Osteoarthritis

Osteoarthritis is considered to be the most common joint disease [32], characterized by progressive degradation of articular cartilage, associated with remodeling of other tissues in the synovial joints and resulting in joint malfunction, pain and disability [33]. Although OA pathogenesis is still unclear, most scientists agree that it represents a complex degenerative disease caused by the interaction of many genetic and environmental factors, such as advanced age, tissue and cellular damage, obesity, joint overuse, and genetic susceptibility [34,35,36]. Several molecular analyses have shown that OA development is linked to genetic variability; genome-wide association studies and quantitative trait analysis have identified many candidate genes within loci associated with increased risk of OA hip and knee [37,38]. However, interesting recent data are emerging from the study of epigenetic regulatory mechanisms whole blood and in tissues mainly affected in OA patients (cartilage, subchondral bone, synovium) [39] (Table 2).

### 4.1. Whole Blood 

The severity of OA is often overlooked, partly due to the lack of soluble diagnostic and prognostic biomarkers that would allow OA to be identified before the appearance of radiographic signs and pain development. Analysis of extracellular genomic material such as miRNAs, lncRNAs, snoRNAs, mRNAs and cell-free DNAs has allowed the identification of aberrantly expressed factors in the body fluids of OA patients that could represent novel disease biomarkers [52]. However, DNA methylation patterns in blood may also have a great potential as non-invasive biomarkers by modulating the expression of genes involved in OA pathogenesis. One of the first reported pilot studies on this topic investigates the possibility of predicting future radiographic progression of knee osteoarthritis on the basis of the epigenetic signature in blood [40]. A DNA methylation study was performed by using Illumina Infinium HumanMethylation450k or 850k arrays on 58 future radiographic progressors compared (cases) to 58 non-progressors (controls). The data obtained have been used to develop a DNA methylation-based predictive method useful for discriminating between case and control groups, suggesting that methylation in Peripheral Blood Mononuclear Cells (PBMCs) could represents a useful biomarker of radiographic progression in OA patients [40]. Based on these data, an epigenome-wide cross-tissue correlation study was performed to characterize the DNA methylation pattern, with the aim of understanding whether blood can be considered a valid bone-surrogate [41]. The analysis identified 28,549 CpG sites with similar degrees of methylation in both tissues, and some common genes implicated in bone metabolism, including *Engrailed Homeobox 1 (EN1)*, *Estrogen Receptor 1 (ESR1)*, *Wnt Family Member 16 (WNT16)* and *RANKL*. Overall these results demonstrate that peripheral blood can reflect bone methylome and capture sites related to bone regulation [41].

### 4.2. Cartilage Tissue

Much work has been done over the past several years linking epigenetic changes in cartilage tissue with OA. Targeted methylation analyses identified significant alterations in genes belonging to the matrix metalloproteinases (MMPs) family (*MMP3, MMP6, MMP9, MMP13, ADAMTS4*), as well as in the obesity- and inflammation-linked leptin genes (*IL1β, NOS2, GDF5, SOD2, SOX9*) [53,54,55,56,57,58,59,60]. Interestingly, novel emerging data also identified aberrant methylation patterns associated with miRNA and lncRNA-encoding genomic regions, expanding the landscape of mechanisms involved in OA pathogenesis. Methylation-specific PCR (MS-PCR) of *Tissue Inhibitor of Metalloproteinase 3* (*TIMP-3*) gene in cartilage samples from 15 OA patients and 7 healthy subjects showed an increased degree of methylation level in the *TIMP-3* promoter region. Functional studies unraveled a regulatory mechanism induced by the X-Inactive Specific Transcript (XIST) lncRNA capable of recruiting DNMT1, DNMT3A and DNMT3B enzymes that catalyze the addition of CH3 groups on *TIMP-3* promoter cytosines. This evidence suggests that TIMP-3 and lncRNA XIST might represent two novel epigenetic targets in OA pathogenesis [42]. The role of ncRNAs has been further supported by the study from Zhang et al. investigating the methylation levels of *miR34a* gene, a known regulator of apoptosis in OA rats, in chondrocytes from OA patients and healthy subjects. This study identified a novel interaction between miR34a and the Small Nucleolar RNA Host Gene 9 (SNHG9). SNHG9 overexpression can induce downregulation of miR34a through DNA hypermethylation, thereby decreasing the apoptosis of chondrocytes in OA patients [43]. In addition to gene-specific DNA methylation analysis, a variety of genome-wide approaches using DNA methylation arrays have also been reported [61,62,63,64,65]. Recently, Alvarez–Garcia and colleagues demonstrated that normal and OA knee articular cartilage can be distinguished based on different DNA methylation profiles [44]. They identified several differentially methylated CpG sites that comprise a total of 500 genes, revealing that healthy and OA knee articular cartilages exhibit substantially different methylomes. Several transcription factors are hypermethylated (ATOH8, MAFF, NCOR2, TBX4, ZBTB16 and ZHX2) with reduced expression in OA cartilage. TBX4 is involved in the regulation of developmental processes and is required for muscle and tendon morphogenesis. ZBTB16 enhances the expression of Sox9, which is the master regulator of the chondrogenesis process. These findings suggested that methylation-related changes in key transcription factors represent an important mechanism that may explain changes in chondrocyte transcriptome and function in OA [44]. In agreement with these data, Zhao et al. discovered the existence of three hypomethylated genes (*Tumor Necrosis Factor Receptor-Associated Factor 1, TRAF1; Connective Tissue Growth Factor, CTGF; Chemokine (C-X3-C) Ligand 1, CX3CL1*) in OA chondrocytes, inversely correlated with mRNA expression [45]. In more a recent study, the role of enhancers has also been explored by the quantification of CpG methylation in 108 samples from patients with hip and knee OA with respect to healthy individuals [46]. A total of 8111 differentially methylated CpGs from enhancer regions were identified, confirming the importance of DNA methylation changes in both types of OA. In addition, a comparison of enhancer methylation levels between knee and hip OA revealed differences in source-dependent patterns. These results indicate that aberrant enhancer methylation is linked to OA phenotypes and that a comprehensive atlas of enhancer methylation could be an interesting tool both for further investigation of these mechanisms and for drug development [46].

### 4.3. Subchondral Bone

Although cartilage degeneration is the main feature of OA, increasing evidence has shown that the underlying subchondral bone plays a critical role in disease initiation and progression [66,67,68]. It has been reported that the presence of subchondral bone stiffness can induce a reduction in its viscoelastic properties as well as a loss in shock absorption capacity, thus causing significant additional mechanical loading and subsequent breakdown of the overlying cartilage. Conversely, damage to the cartilage may adversely affect the adjacent subchondral bone, triggering a pathogenic circle in the OA joint [66]. Recently, Jeffries et al. performed the first EWAS by using Illumina Human Methylation 450 arrays to characterize DNA methylation patterns in subchondral bone, along with eroded and intact cartilage, from 12 patients with hip OA [47]. Profiling analysis identified 7316 differentially methylated CpG sites, mostly hypomethylated, in subchondral bone underlying eroded cartilage and 1397 sites in the overlying eroded cartilage, which in total showed 126 shared sites [47]. This analysis identified new genes related to OA pathogenesis, including the most highly hypomethylated gene *EIF2C2*, encoding for the protein Argonaute 2, which is a key member of RISC complex and processes small interfering RNAs [69]. *TGFB3* also showed a significant hypermethylation pattern; this gene encodes a secreted ligand of the TGF-β protein superfamily that plays an important role in chondrogenesis and endochondral bone formation, as well as in mesenchymal cell proliferation and angiogenesis [70,71]. Surprisingly, four known susceptibility genes in OA were identified sharing the same methylation pattern in both OA subchondral bone and OA cartilage: *Insulin-Like Growth Factor-Binding Protein 7 (IGFBP7)*, *Low-Density Lipoprotein Receptor-Related Protein 5 (LRP5)*, *FTO* and *Nuclear Receptor Corepressor 2 (NCOR2)*. These results identify new genes related to OA phenotype and suggest the existence of similar epigenetic mechanisms in both underlying subchondral bone and overlying eroded cartilage in OA patients [47]. Similarly, Zhang et al. examined, by EWAS, 12 knee joints in order to characterize distinctive features of OA disease progression associated with epigenetic alterations in subchondral bone [48]. Three regions on the tibial plateau representing early, intermediate, and late stages were studied. Significant Differential Methylated Probes (DMPs) and Differential Methylated Genes (DMGs) were identified in all three groups of analysis. These DMPs showed different methylation patterns in cartilage and subchondral bone, suggesting the tissue-specific role of DNA methylation during disease progression. Furthermore, comparison of the identified DMGs indicated that DNA methylation changes occurred earlier in subchondral bone, preceding those in cartilage [70]. These data suggest that methylation represents a key mechanism for the differential regulation that is detected between cartilage and subchondral bone [48]. Thus, in agreement with other experimental evidence, differential methylation represents a key molecular hallmark to distinguish cartilage and subchondral bone.

### 4.4. Synovium 

Synovial membrane plays a key role in chronic joint inflammation, neovascularization and cartilage destruction, a pathological condition that requires the involvement of macrophage-derived pro-inflammatory cytokines, such as IL1β, Interleukin-6 (IL-6), Interleukin-8 (IL-8) and Tumor Necrosis Factor-alpha (TNF-α) [72,73,74,75]. In a skeletal system, IL-6 activates osteoclasts and stimulates synovium to produce MMPs, which are responsible for cartilage degradation in OA, suggesting that blocking IL-6 overexpression in synovial fibroblasts may prevent OA progression [76]. Recent studies attempt to clarify the molecular mechanisms underlying IL-6 overproduction in OA patients by analyzing epigenetic signatures. Yang et al. isolated synovial fibroblasts overexpressing *IL-6* from OA patients to investigate epigenetic modifications occurring in the *IL-6* promoter region, such as DNA methylation, histone acetylation and histone methylation [49]. For this purpose, the binding of Methyl-CpG Binding Protein 2 (MeCP2), DNMT1, DNMT3a, Histone Deacetylase 1 (HDAC1), Histone Acetyltransferase 1 (HAT1), CREB Binding Protein (CBP) and p300 on the *IL-6* promoter region were studied. Results showed a hypomethylation pattern and histone hyperacetylation, as well as weaker binding of MeCP2, Dnmt3a and HDAC1 in OA patients compared to healthy subjects, confirming the involvement of epigenetic changes in *IL-6* promoter at the synovial level in OA pathophysiology [49]. In recent years, small non-coding RNAs, such as miRNAs, have been identified in synovial fluid and it has been shown that transcriptional silencing of these factors due to DNA hypermethylation occurs in several pathological conditions, including OA disease. *MiR-140-5p* is particularly expressed in articular cartilage and is upregulated during chondrogenesis [77]; furthermore, its expression was found to be reduced in knee OA synovial fluid and articular chondrocytes [78,79,80]. In contrast, *miR-146a* is deregulated in OA and its expression pattern correlates with inflammatory responses in articular cartilage and synovial tissues from OA patients [81,82]. Based on this scientific evidence, Papathanasiou and colleagues performed a DNA methylation analysis of *miR-140-5p* and *miR-146a* genes in chondrocytes and synoviocytes from OA patients [50]. Data obtained identified a hypermethylation pattern of both the *miR-140-5p* regulatory region and *miR-146a* promoter. The percentage of methylation reflected a strong negative correlation with *miR-140-5p* and *miR-146a* expression. In synoviocytes, *miR-146a* gene hypermethylation was responsible for the reduced binding affinity of the NF-kB transcription factor, confirming the inflammatory role of *miR-146a* in disease progression and suggesting a methylation-based *miR-146a* approach as a new therapeutic strategy for OA patients [50]. Finally, Zhang and colleagues performed in vitro functional assay followed by MSP-PCR to analyze the epigenetic control of the *miR-130a* gene, a promoter of chondrocyte proliferation, in synovial fluid from OA patients and healthy subjects [51]. The results showed that *C-Terminal Binding Protein 1-Antisense RNA 2* (*CTBP1-AS2*) was upregulated in OA and inversely correlated with *miR-130a*. In addition, in chondrocytes of OA patients, overexpression of *CTBP1-AS2* led to increased methylation of the *miR-130a* gene and downregulated expression of miR-130a [51]. Analysis of chondrocyte proliferation showed that overexpression of *miR-130a* led to an increased proliferation rate of OA chondrocytes, whereas overexpression of *CTBP1-AS2* led to a decreased proliferation rate of chondrocytes and reversed the effects of overexpressing *miR-130a*. Therefore, the upregulation of *CTBP1-AS2* may increase the methylation of miR-130a gene to inhibit chondrocyte proliferation in OA tissues. 

## 5. An Epigenetic Link between Osteoporosis and Osteoarthritis Bone-Related Phenotypes

The identification of shared risk factors between OP and OA led researchers to hypothesize a genetic/epigenetic background common for both diseases triggering to overlapping pathogenic mechanisms [83]. For the first time, Delgado–Calle and colleagues investigated the role of DNA methylation in human bone from OP and OA patients by analyzing two key factors in bone metabolism: *RANKL* and its soluble decoy receptor *OPG*. Two *RANKL* CpG islands: upstream and downstream of the TSS, were analyzed, but despite significant differences in gene expression between the two pathological groups, no differences in *RANKL* methylation pattern were detected. Moreover, methylation analysis in a specific CpG island near the TSS of *OPG* gene compared to the gene expression level showed no statistically significant differences in either disease group. It is therefore possible that additional mechanisms, other than DNA methylation, could play a role in modulating RANKL and OPG expression levels [84]. Later, the same research team performed a EWAS profiling analysis in trabecular bone from 27 patients with OP hip fractures and 26 patients with OA hip, identifying 241 CpG sites located in 228 genes, with significant differences in methylation patterns between the two disease groups. However, these differentially methylated regions of the genome are not located within genes directly involved in osteoblast and osteoclast activity, though network analysis identified key genes associated to bone metabolism, such as *collagen*, *alkaline phosphatase* and *NF-KB*. This result led us to hypothesize that although bone cells do not appear to be directly regulated by epigenetic mechanisms, upstream regulatory genes could use these same mechanisms to modulate their activity [85]. More recently, Li et al. analyzed spongy bone samples from 12 individuals divided into four experimental groups: osteoporotic (OP), osteoarthritis (OA), osteoporotic and osteoarthritis (OP&OA) and healthy (N) subjects. The MeDIP-chip study revealed increased methylation levels in OP and OA groups compared to OP&OA group at a total of 1222 different sites. In addition, four common genes (*Peptidylprolyl Isomerase Like 3, PPIL3; NGG1 Interacting Factor 3 Like 1, NIF3L1; Smoothelin, SMTN; Calcium Homeostasis Modulator Family Member 2, CALHM2*) in OP and OA groups were identified [86]. These genes might be involved in the development and progression of OP and OA aging-bone diseases by representing a link between these two pathological conditions. The investigation of common epigenetic regulatory mechanisms could be a crucial step towards diagnosis and treatment related to bone metabolism diseases.

## 6. On the Road of Epigenetic Therapies for Bone Diseases

The reversible nature of epigenetic alterations has induced the development of therapeutic strategies targeting various epigenetic components to treat metabolic diseases such as OP and OA.

Pharmacological agents targeting DNA methylation have been already demonstrated to be effective in different anticancer therapies, while also holding great promise for treatment of degenerative bone metabolic pathologies. DNMTs are the most studied epigenetic targets for pharmacological intervention and DNMT inhibitors (DNMTi) are considered the most powerful compounds to revert abnormal DNA methylation and restore a normal profile. The nucleoside-mimic compounds 5-Azacytidine (5-Aza-CR) and 5-aza-2′-deoxycitidine (5-Aza-CdR) are two powerful DNMTi already used as epigenetic drugs for the treatment of myeloid malignancies [87]. Their successful clinical utilization in cancer therapies induced us to evaluate their potentiality in therapeutic strategy for intervening in OP [88] and OA [89] epigenetic landscapes. Indeed, in vitro studies have demonstrated the ability of 5-Aza-CR to increase the expression of bone metabolism-related genes by enhancing osteogenic differentiation of MSCs [90,91]; 5-Aza-CdR has been found to promote a switch from adipogenesis to osteoblastogenesis in 3T3-L1 preadipocyte by activating the Wnt signaling pathway [92] and a recent study has indicated its ability to inhibit titanium-induced osteolysis in mice by regulating the RANKL/OPG ratio [93]. Natural products, acting as DNMT inhibitors, have been also tested since cytidine analogues require incorporation into DNA and might cause cytotoxicity [94]. In particular epigallocatechin-3-gallate (EGCG), a polyphenolic compound with DNMT inhibition activity, has been shown to protect against secondary osteoporosis in a mouse model via the Wnt signaling pathway [95]. Although the above-mentioned DNMTi result in a promising approach for metabolic bone disorders therapeutics, all these are not selective inhibitors of DNA methylation and possible side effects are the main problem for their clinical use. To overcome this problem, it will be necessary to better understand specific epigenetic patterns in genes responsible for bone metabolic diseases in order to design selective inhibitors for a specific DNMT isoform [96] minimizing side effects in future therapeutics approaches.

## 7. Concluding Remarks

DNA methylation is the most widely studied epigenetic mechanism regulating biological processes through modulation of gene expression. This systematic review aimed to provide an up-to-date overview of DNA methylation studies conducted in patients with both OP and OA bone diseases, distinguishing the modulatory sites identified in the different affected tissues. Given the multiplicity of target tissues involved in these metabolic bone pathologies, it is crucial to investigate the multiple mechanisms regulating the pathogenesis of OP and OA diseases, in both systemic and localized contexts. The emerging data presented herein demonstrate a growing interest in the study of DNA methylation as a component of epigenetic susceptibility to OP and OA. However, further functional studies are still necessary to bridge the knowledge gaps regarding the bone-specific mechanisms of DNA methylation and its association with clinical phenotypes. In particular, tissue-specific role of methylation in OP and OA aetio-pathogenesis have to be deeply investigated. Bone-associated epigenomes might represent excellent diagnostic and prognostic biomarkers in OA and OP and pave the way to identifying novel potential therapeutic targets in age-related bone metabolic diseases.

## Figures and Tables

**Table 1:** Summary of DNA Methylation Studies in Osteoporosis.

Tissue	Sample	Technique	Analysis Target	Analysis Outcome	Reference
Whole blood	323 postmenopausal women	Combined bisulfite restriction analysis (COBRA)	Regulatory region of the Alu sequence	Alu hypomethylation in individuals with lower bone mass and older age	[16]
	24 OP patients, 24 healthy subjects	Bisulfite specific PCR (BSP)	CpG island in promoter region of *BMP2* gene	*BMP2* hypermethylation in OP patients	[17]
	30 OP patients, 30 healthy subjects	Methylation sequencing	CpG island in promoter region of *TBCF1D8* gene	*TBCF1D8* decreased methylation in OP patients	[18]
	22 postmenopausal OP women, 22 healthy women	Infinium human methylation 450 K and Pyrosequencing	Genome-wide analysis and validation of five genes involving in bone biology	*ZNF267* hypomethylation, *ABLIM2*, *RHOJ*, *CDKL5* and *PDCD1* hypermethylation in OP patients	[19]
	32 OP patients, 16 healthy subjects	Infinium Human-Methylation450 BeadChips	Genome-wide DNA methylation analysis	DNA methylation patterns of blood do not reflect OP	[20]
Bone tissue	27 OP patients, 36 healthy subjects	Infinium Human-Methylation450 BeadChip	CpG island in promoter region of *SOST* gene	Increased CpG methylation in OP patients	[21]
	12 postmenopausal OP women, 8 healthy subjects	Bisulfite specific PCR (BSP)	CpG island in promoter region of *SOST* gene	Increased CpG methylation in OP patients	[22]
	16 OP patients, 16 healthy subjects	Bisulfite Sequencing	CpG island in promoter region of *SOST* gene	*SOST* gene promoter was slightly demethylated in OP	[23]
	84 postmenopausal OP women	Infinium human methylation 450 K and Pyrosequencing	Genome-wide DNA methylation profiles	*MEPE*, *SOST*, *DKK1*, *WIF1* correlate with the highest number of methylated CpGs	[13]
	16 OP patients, 16 healthy subjects	Bisulfite Sequencing	OPG/RANKL	*RANKL* hypomethylation and *OPG* hypermethylation in OP patients	[24]
	5 postmenopausal OP women, 3 healthy subjects	Illumina 850K methylation microarray analysis	Genome-wide DNA methylation profiles	13 differentially methylated genes: *PLEKHA2*, *PLEKHB1*, *PNPLA7, SCD, MGST3, TSNAX, PRKCZ, GNA11, COL4A1, SOX6, ACE, SYK* and *TGFB3*	[25]

**Table 2:** Summary of DNA Methylation Studies in Osteoarthritis.

Tissue	Sample	Technique	Analysis Target	Analysis Outcome	Reference
Whole blood	58 OA radiographic progressors, 58 OA radiographic nonprogressors	Illumina Infinium HumanMethylation450k/850k arrays	Genome-wide analysis	DNA methylation-based models of PBMCs are predictive of OA radiographic progression	[40]
	12 Caucasian women with OA	Epigenome-wide cross-tissue correlation study	Genome-wide analysis	*EN1, ESR1, Wnt16* and *RANKL* similarly methylated in blood and bone tissue	[41]
Cartilage tissue	15 OA patients, 7 healthy subjects	Methylation-specific PCR (MS-PCR)	Promoter region of *TIMP-3* gene	Increased methylation level in *TIMP-3* promotor region	[42]
	60 OA patients, 60 healthy subjects	Methylation-specific PCR (MS-PCR)	*miR-34a* gene	Increased methylation level in *miR-34a* gene	[43]
	12 OA patients, 11 healthy subjects	Infinium HumanMethylation450 BeadChip methylation array	DNA methylation	DNA methylation profiling revealed 929 differentially methylated sites, with a total of 500 genes	[44]
	25 OA patients, 12 healthy subjects	Illumina HumanMethylation 450 arrays	Genome-Wide DNA Methylation Study	Decreased methylation of *TRAF1, CTGF, CX3CL1* genes	[45]
	78 OA patients, 19 healthy subjects	Illumina’s Infinium HumanMethylation450 BeadChip	Genome-Wide DNA Methylation Study	16,816 differentially methylated CpGs, of which 8111 were from enhancers	[46]
Subchondral bone	12 OA patients	Illumina HumanMethylation 450 arrays	Genome-Wide DNA Methylation Study	7316 differentially methylated CpG sites	[47]
	12 OA patients	HumanMethylation 450 BeadChip	Genome-wide DNA methylation profiles	DNA methylation changes in subchondral bone are time-varying	[48]
Synovium	20 OA patients, 15 healthy subjects	Bisulfite sequencing PCR	CpG island in promoter region of *IL-6* gene	*IL-6* hypomethylation in OA patients	[49]
	20 OA patients, 15 healthy subjects	Bisulfite sequencing PCR	*miR-140* and *miR-146a* genes	Hypermethylation of *miR-140* and *miR-146a* genes	[50]
	62 OA patients, 60 healthy subjects	Methylation-specific PCR (MS-PCR)	*miR-130a* gene	Increase of *miR-130a* gene	[51]

## Data Availability

No new data were created or analyzed in this study. Data sharing is not applicable to this article.
